# Dynamics of facial actions for assessing smile genuineness

**DOI:** 10.1371/journal.pone.0244647

**Published:** 2021-01-05

**Authors:** Michal Kawulok, Jakub Nalepa, Jolanta Kawulok, Bogdan Smolka

**Affiliations:** Faculty of Automatic Control, Electronics and Computer Science, Silesian University of Technology, Gliwice, Poland; National Institutes of Health, UNITED STATES

## Abstract

Applying computer vision techniques to distinguish between spontaneous and posed smiles is an active research topic of affective computing. Although there have been many works published addressing this problem and a couple of excellent benchmark databases created, the existing state-of-the-art approaches do not exploit the action units defined within the Facial Action Coding System that has become a standard in facial expression analysis. In this work, we explore the possibilities of extracting discriminative features directly from the dynamics of facial action units to differentiate between genuine and posed smiles. We report the results of our experimental study which shows that the proposed features offer competitive performance to those based on facial landmark analysis and on textural descriptors extracted from spatial-temporal blocks. We make these features publicly available for the UvA-NEMO and BBC databases, which will allow other researchers to further improve the classification scores, while preserving the interpretation capabilities attributed to the use of facial action units. Moreover, we have developed a new technique for identifying the smile phases, which is robust against the noise and allows for continuous analysis of facial videos.

## Introduction

Facial expressions are the observable temporal alterations in human face appearance caused by motions of the muscles located just under the facial skin, controlled with the facial nerve. While there is no doubt that the primary function of facial expressions for humans is to convey information on the emotional state of an individual, their origin from the evolutionary perspective could be quite different [1], for example related with increasing or decreasing the sensory exposure [2]. Facial expression recognition is an inherent capability of humans, and it plays a substantial role in their interpersonal communication. Automatic recognition of facial expressions from digital images and videos has been explored for years, becoming a multidisciplinary research topic that embraces computer vision, machine learning, psychology, neuroscience, and cognitive sciences. Potential applications of recognizing facial expressions are related with healthcare, surveillance, animation engines, driver safety, creating responsive human-computer interfaces, and more [3].

An important direction in facial expression analysis is concerned with assessing the genuineness of the manifested non-verbal messages. In particular, the problem of discriminating between spontaneous and posed smiles has been given considerable attention in the literature [4–6]. Smiles are one of most common facial expressions, and their detection using computer vision techniques has been widely investigated [7]. Over the years, a variety of benchmark datasets were created, including the famous UvA-NEMO Smile Database [4] which contains over a thousand videos with genuine and posed smiles. This encouraged the researchers to focus on recognizing smile genuineness, and the study reported in this paper addresses this interesting problem as well.

### Facial action coding system

Current state of the art in automatic facial expression recognition originates from the work of Ekman and Friesen, who introduced Facial Action Coding System (FACS) [8] to describe the facial activity. In FACS, all the observable expressions are represented as a combination of basic visually discriminable muscle actions, termed *Action Units* (AUs). Importantly, FACS is a descriptive system, which considers the face from an observer’s perspective, rather than performing anatomical or emotional analysis. This makes FACS particularly useful in creating computer vision solutions aimed at recognizing facial expressions from images or videos, as the analysis can be performed in a two-stage approach [9]—first, the AUs are automatically detected, and subsequently their interpretation is performed during the second stage. There have been many successful attempts to exploit FACS for recognizing facial expressions [10, 11], and the mapping between FACS and expressed emotions was confirmed by Wegrzyn et al. in their recent study [12]. Furthermore, Khorrami et al. reported an interesting observation that the features elaborated automatically using deep learning employed for recognizing facial expressions are highly correlated with the AUs defined in FACS [13], which once again confirmed the adequacy of this observation model. Importantly, detection of AUs, alongside assessing their intensity, can be effectively performed relying on computer vision solutions [14–17], and a number of implementations are publicly available.

The dynamic process of manifesting a smile is composed of three main phases, namely: (*i*) *onset* (when the face alters from neutral expression to a smile), (*ii*) *apex* (when the observable expression of the face is a smile with varying intensity), and (*iii*) *offset* (when the facial expression turns back to neutral). A smile is mainly concerned with the following AUs: AU6 (cheek raiser) and AU12 (lip corner puller), however different AUs are very often involved as well. One of the reasons is that there are a wide range of possible underlying emotional states which could be expressed with a smile, including happiness, enjoyment, pleasure, embarrassment, sadness, or even fear, depending on the context. Although the subtle differences between these types of smiles can be relatively easily perceived by humans in most cases (this appears non-trivial for patients with mental disorders, e.g., schizophrenia [18]), it is a challenging computer vision and pattern recognition task. Discriminating between genuine (spontaneous) and posed smiles, along with understanding which facial features exhibit overwhelmingly different human intensions became a vital topic and attracted attention in many domains, ranging from machine learning to clinical research [19]. A more general problem of recognizing the genuineness of manifold facial expressions was recently studied by Healey et al. [20]. They used average intensity of AUs to differentiate between spontaneous and intentionally expressed reactions to positive and negative images. For intentional expressions, the AU intensity was higher both for AUs associated with negative (AU1, AU2, AU4, and AU5) and positive (AU6 and AU12) emotions. However, neither the dynamics of AUs, nor their mutual relation were studied in that research.

### Contribution

Despite many successful attempts to exploit FACS for recognizing facial expressions, AUs are not commonly used for assessing smile genuineness. The only attempt to exploit AUs for automatic recognition of spontaneous smiles was reported in 2006 by Valstar et al. [21]. Three AUs related with the eyebrow movements (AU1, AU2 and AU4) were studied in [21]. Recently, Ruan et al. [22] reported a psychological study aimed at improving the people’s ability to differentiate between posed and spontaneous smiles by focusing on AU6 and AU12 related with the mouth movements. The recent approaches are either based on direct analysis of facial landmarks [4], they rely on spatial-temporal textural features [5], or are underpinned with the features extracted from smile intensity dynamics [6].

The goal of the research reported here was to verify whether AUs defined in FACS contain sufficient information to discriminate between posed and spontaneous smiles, as this problem has not been tackled in the literature so far. We explore how to exploit AUs for recognizing smile genuineness, to increase the interpretability of automated methods that solve this task. Furthermore, we report our study to investigate which AUs carry most valuable information in assessing whether a smile is posed or spontaneous. Overall, our contribution is threefold:

1. We introduce the AU *Dynamics Analysis* (AUDA) method for recognizing smile genuineness. The method is underpinned with new features (we publish the AUDA features extracted for the UvA-NEMO and BBC benchmarks (https://doi.org/10.7910/DVN/X5QGLA), which should allow for further research focused on improving their classification) that capture the dynamics of particular AUs, as well as their mutual relations.
2. We study the relevance of particular AUs, as well as the pair-wise differences in their dynamics, for deciding whether an observed smile is spontaneous.
3. We propose a new approach towards detecting the smile phases (the source code for detecting the smile phases is available at https://github.com/jkawulok/audaphases). In contrast to many existing approaches, we do not assume that a given video sequence presents a single cycle of a smile composed of onset, apex and offset, making it suitable for continuous face analysis.

The results of our experimental study indicate that the proposed features have competitive discriminating capabilities when compared with the features exploited by the existing state-of-the-art techniques [4, 23]. At the same time, their physiological interpretation is straightforward, as they are entirely based on the AUs. This showcases that the FACS features convey the information that allows for discriminating posed smiles from spontaneous ones.

### Related work

#### Facial expression recognition

Analysis and recognition of facial expressions has been intensively studied in the literature [9, 11, 24–26]. Existing approaches are either based on the *holistic* features, extracted from the entire facial region, or on the *local* ones retrieved from particular facial components and facial landmarks. Furthermore, the features can be extracted from the spatial domain [27, 28] (each image is analyzed independently) or directly from the spatial-temporal domain [29] (the features are extracted across multiple frames of a video sequence).

Taking into account whether and how FACS is exploited, two approaches can be distinguished: (*i*) to detect AUs given a still facial image (or an image sequence) followed by interpreting the recognized actions [30], and (*ii*) to recognize the expressions or non-verbal messages directly from the facial region without detecting the AUs [31]. The latter approach encompasses both local and global features, including Local Binary Patterns (LBPs) [32], Gabor wavelets [33, 34], extreme learning machines [7], and many solutions based on deep Convolutional Neural Networks (CNNs) [35, 36]. Moreover, some of the recent methods based on deep learning exploit the knowledge on FACS in an indirect way. Khorami et al. studied the deep features learned by CNNs trained to recognize facial expressions, and they discovered that these features resemble the AUs defined in FACS [13]. Furthermore, Liu et al. proposed a deep network [37], whose architecture is inspired by the AUs. In this way, the analysis is intended to be split into detecting the AUs using adaptive receptive fields, and then the network groups the features to recognize specific expressions.

#### Detection of facial action units

The problem of detecting AUs from face images has been recently thoroughly reviewed by Martinez et al. [25]. The general pipeline for detecting AUs encompasses three main phases, namely: *preprocessing* aimed at detecting face alongside the facial landmarks, topped with face normalization, which is followed by *feature extraction* to prepare the basis for higher-level *analysis of facial actions* to detect, recognize and classify the particular AU.

Face and facial landmark detection has been widely explored [38] and among most effective approaches are active appearance models [39], supervised descent [40], or constrained local model [41], whose implementation is available in the OpenFace suite [42, 43] (OpenFace library is available at https://cmusatyalab.github.io/openface). From the detected landmarks, local appearance-based features, with different variations of LBPs [44] and Histogram of Oriented Gradients (HOG) [45] being most common, are extracted and classified to detect particular AUs. In OpenFace, the geometry-based features are coupled with HOG features reduced using Principal Component Analysis (PCA), and classified with a linear Support Vector Machine (SVM) to detect the AUs [46]—recently, in [47], this SVM-HOG approach was reported to outperform solutions based on CNNs.

There have also been some successful attempts to detect AUs using CNNs [48]—the most important challenge here consists in the need for large amounts of annotated data. Tong et al. reported to increase the accuracy of detecting AUs by exploiting their dynamic and semantic relationships [16]. Relationship between the manifested AUs have been also recently studied by Wang et al. [49] and it was subsequently exploited to improve their recognition using a hybrid Bayesian network. Overall, state of the art in AU detection allows for excellent performance for frontal faces in controlled environment, and the main research challenges are concerned with robustness against head pose variations and realistic illumination conditions. Importantly, the algorithms for facial expression recognition that are underpinned with AU detection are easier to interpret and understand.

#### Smile genuineness

Discrimination between posed (deliberate) and spontaneous (genuine) smiles from facial images and videos is an intensively explored research topic [50, 51]. In the last decade, there have been many advances made focused both on developing new computer vision techniques, as well as creating appropriate databases that could serve as benchmarks, including the excellent UvA-NEMO Smile Database [4]. The latter task is particularly important, as it is quite challenging to ensure that the person being recorded is presenting the expected (i.e., posed or spontaneous) smile [52]—creating such benchmarks requires close cooperation between psychologists, camera operators, and computer vision specialists. Overall, the process of collecting such data remains an important challenge in expression genuineness recognition.

Most of the state-of-the-art algorithms for recognizing spontaneous and posed facial behaviors are focused on the temporal analysis of various facial features. In one of the earliest approaches towards recognizing smile genuineness, Cohn and Schmidt [53] investigated changes in the Smile Onset Amplitudes and their Durations (SOAD), extracted from detected and tracked facial landmarks, to find a strong evidence that spontaneous smiles are characterized by smaller amplitudes and significantly more stable relations between these two features. Valstar et al. [21] exploited the AUs focused on the eyebrow region (i.e., AU1, AU2, and AU4), extracted from the positions of facial landmarks. An interesting, yet simple approach, in which the asymmetry of facial expressions is exploited, was presented by Senechal et al. [54]. Extracting distance-based and angular features from eyelid movements for this task was proposed in [55].

Dibeklioğlu et al. demonstrated that although the eyelid features are most discriminating [4], as claimed in [53], the classification performance can be boosted, if these features are coupled with those extracted from other facial components (encompassing, e.g., cheeks and/or lip corners). This finding indicates that different facial regions can contribute differently to the classification of smiles in their particular phases. Here, the onset phase is detected as the longest continuous increase in the distance between the mouth corners, the offset is the longest continuous decrease, and the frames between these two are considered to represent the apex phase. Such an approach is not robust against inaccurate localization of facial features, and it is underpinned with the assumption that a given sequence always presents a single smile cycle. In order to address the shortcoming resulting from the sensitivity to facial feature localization, appearance-based techniques were also developed. Liu and Wu proposed to detect AU6 and AU12 using Gabor wavelets with 2D PCA and Adaboost, and final classification to assess smile genuineness is performed using SVM [56]. Recently, the psychological aspects of focusing on these two AUs while learning people to differentiate between posed and genuine smiles were explored by Ruan et al. [22].

Pfister et al. [57] proposed to utilize the Completed Local Binary Pattern (CLBP)—the standard LBP is complemented with textural features from Three Orthogonal Planes—which creates an appearance-based local spatial-temporal descriptor (CLBP-TOP). The CLBP-TOP descriptor was enhanced by Wu et al. [23]—the entire image sequence is divided into blocks in both spatial and temporal domains, using the flexible facial sub-region cropping. Then, five discriminative facial points (eyes, lip corners, and nose tip) are detected and tracked to retrieve facial sub-region volumes which are further analyzed. Each sub-region volume is divided into three blocks in the temporal domain, reflecting three smile phases: onset, apex, and offset (in a similar manner to [4]). In this paper, we refer to that approach as CLBP-TOP+. In addition to that, the authors in [23] proposed an adaptive learning procedure to extract an optimal (most discriminative) subset of all CLBP-TOP features (termed disCLBP-TOP). Although this algorithm retrieved high classification scores, inaccurate detection of facial landmarks can notably jeopardize its performance. The initial work by Wu et al. was further improved in [5] by introducing a discriminative learning model (DLM) to classify the disCLBP-TOP features.

In our earlier work [6], we proposed to analyze Smile Intensity Dynamics (SID) to estimate smile genuineness. Smile intensity is measured in the facial region, as well as in two facial components—the eyes region and the mouth region. The assessment is made in a frame-wise manner, relying on the LBP features classified with SVM. Dynamics of smile intensity is analyzed in each frame, as well as from the whole sequence, and these features are classified once again using SVM to distinguish the spontaneous from posed smiles.

Overall, the state-of-the-art methods that were reported to render high classification scores do not rely on the AUs. Most of them are based on the features extracted directly from the images or they exploit the landmark locations and smile intensities. This makes it more challenging to integrate these methods with the existing AU-based systems for facial expression analysis.

Smile genuineness recognition may also be performed employing multi-modal techniques which benefit from the observation that people communicate by the means of language, facial expressions, head movement, gestures and poses [58]. To fully exploit the information coming from different sources, the multi-modal methods fuse them to improve the classification performance. This fusion may be performed at various abstraction levels (they are often referred to as *early*, *mid-level*, and *late* fusion strategies), e.g., across different smile phases, or for various facial regions. In [59], three different facial regions are used to extract features (eyes, cheeks, and mouth). Then, SVMs are trained for each region separately, and they are used to classify the feature vectors. The algorithm which fuses head, face, and shoulder modalities was proposed in [60] (different landmark trackers were employed for each modality). The authors efficiently combined these modalities, and highlighted which of them carry discriminative information. According to the authors, the tracked facial landmarks were related with AU6, AU12, and AU13. Another interesting research direction includes thermal imaging, in which the heat radiated from the face is used to recognize deception [61]. Recently, Saito et al. demonstrated that smile genuineness can be assessed based on a signal measured with smart eyewear equipped with 16 photo-reflective sensors [62].

## Method

**Fig 1:**
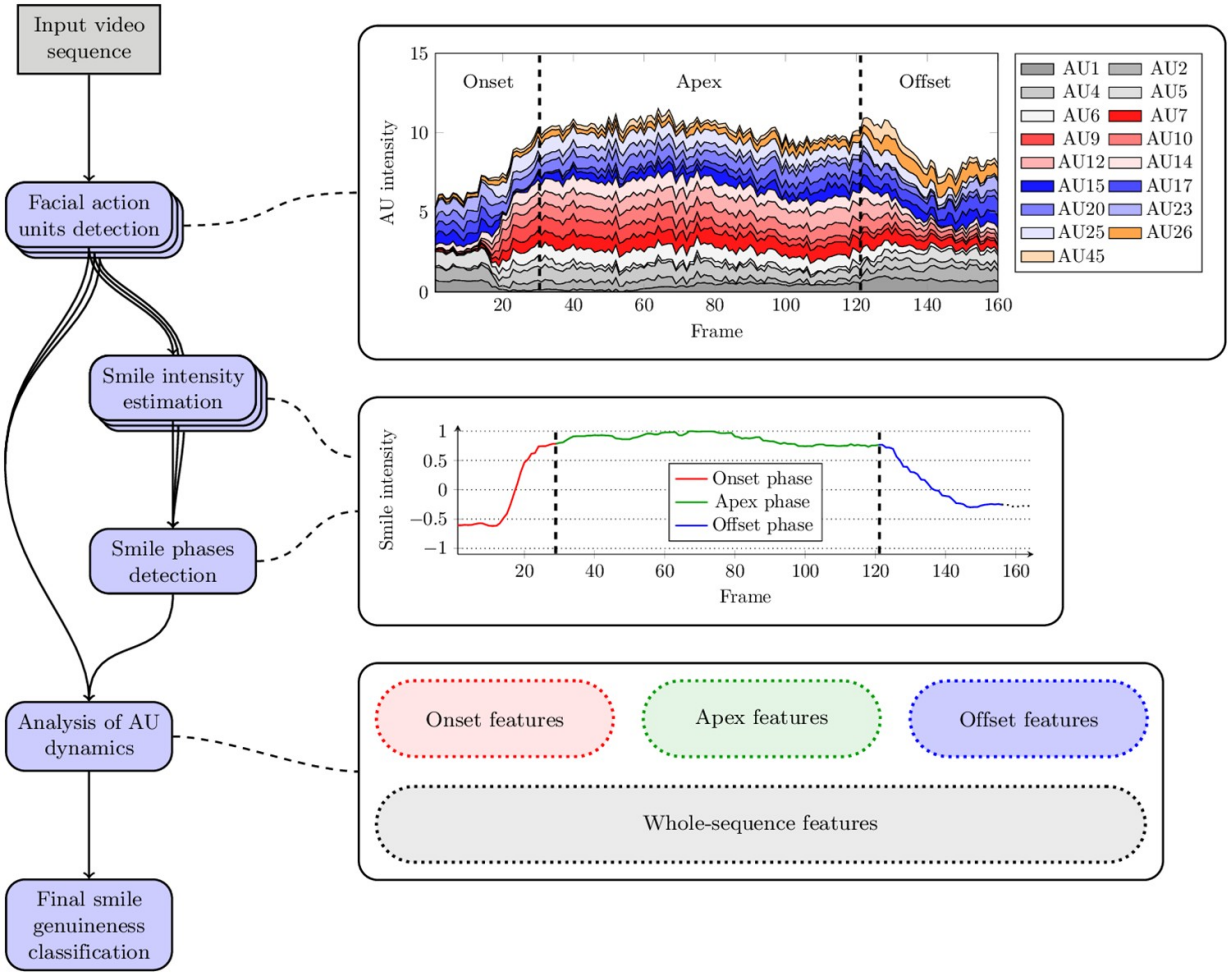
A flowchart presenting the process of classifying a smile as spontaneous or posed based on the AU dynamics. From a video sequence, facial AUs are recognized (in a frame-wise manner, indicated by multiple blocks in the diagram), and the smile intensity is estimated for every frame to determine smile onset, apex, and offset phases. AU dynamics are captured for each individual phase, as well as for the entire sequence, and these feature vectors are eventually classified using an SVM ensemble. The intensities of individual AUs are normalized, hence their sum may theoretically reach the value of 17. The smile intensity is scaled within -1 and 1, the value of 0 being the decision boundary of the classifier. The plots were obtained based on the data extracted from the 020_spontaneous_smile_2 sequence in the UvA-NEMO database.

A general overview of the proposed approach is presented in Fig 1. At first, facial AUs are detected using the SVM-HOG technique [46]—for every frame, the intensity for each of 17 AUs is retrieved (in the plot, the intensities of individual AU are scaled from 0 to 1), which forms a frame-wise AU feature vector. Subsequently, we employ an SVM to estimate the smile intensity from each AU feature vector. The obtained smile intensity series is processed to detect a smile in the temporal domain (here, the smile intensity is scaled between -1 and 1, with the value of 0 being the classifier’s decision boundary between the *smile* and *non-smile* classes) and to divide it into three phases (i.e., onset, apex, and offset). For each detected phase, as well as for all of them, we capture the dynamics of each AU alongside their mutual dependencies, to extract four feature vectors that characterize the considered sequence. Finally, these feature vectors are classified using an SVM ensemble to determine whether the presented smile is spontaneous or posed. These subsequent steps are discussed in detail later in this section.

### Capturing the expression dynamics

In order to detect the smile phases, as well as to extract features which allow for discriminating between posed and spontaneous smiles, we analyze a series (***v***) of estimated intensities (of the smile and/or individual AUs) to capture the dynamics of the signal. For a series of the intensity values {*v*_*i*_}, we first apply median filtering over three consecutive values, followed by linear regression in a sliding window of *ω* subsequent scores with a unit stride. While we assume the series to have a frequency of 50 frames per second (fps), we adjust the window length accordingly for sequences of a different time rate (alternatively, the sequences could be normalized, so that the time span between subsequent intensities in the series equals 20 ms). For each window, we obtain a trend line characterized by its slope:

$$\begin{array}{c} \delta =\frac{\sum_{i=1}^{\omega }(t_{i}-\bar{t})(v_{i}-\bar{v})}{\sum_{i=1}^{\omega }{\left(t_{i}-\bar{t}\right)}^{2}} \end{array}$$ (1)

and regression coefficient:

$$\begin{array}{c} r=\frac{\sum_{i=1}^{\omega }(t_{i}-\bar{t})(v_{i}-\bar{v})}{\sqrt{\sum_{i=1}^{\omega }{\left(t_{i}-\bar{t}\right)}^{2}\sum_{i=1}^{\omega }{\left(v_{i}-\bar{v}\right)}^{2}}}, \end{array}$$ (2)

where *t* is the frame capture timestamp, $\bar{v}$ and $\bar{t}$ are the mean values of *v* and *t* inside the window. The regression coefficient *r* ∈ [−1;1] indicates how well the linear trend fits the data (the higher its absolute value is, the more linear they are).

**Fig 2:**
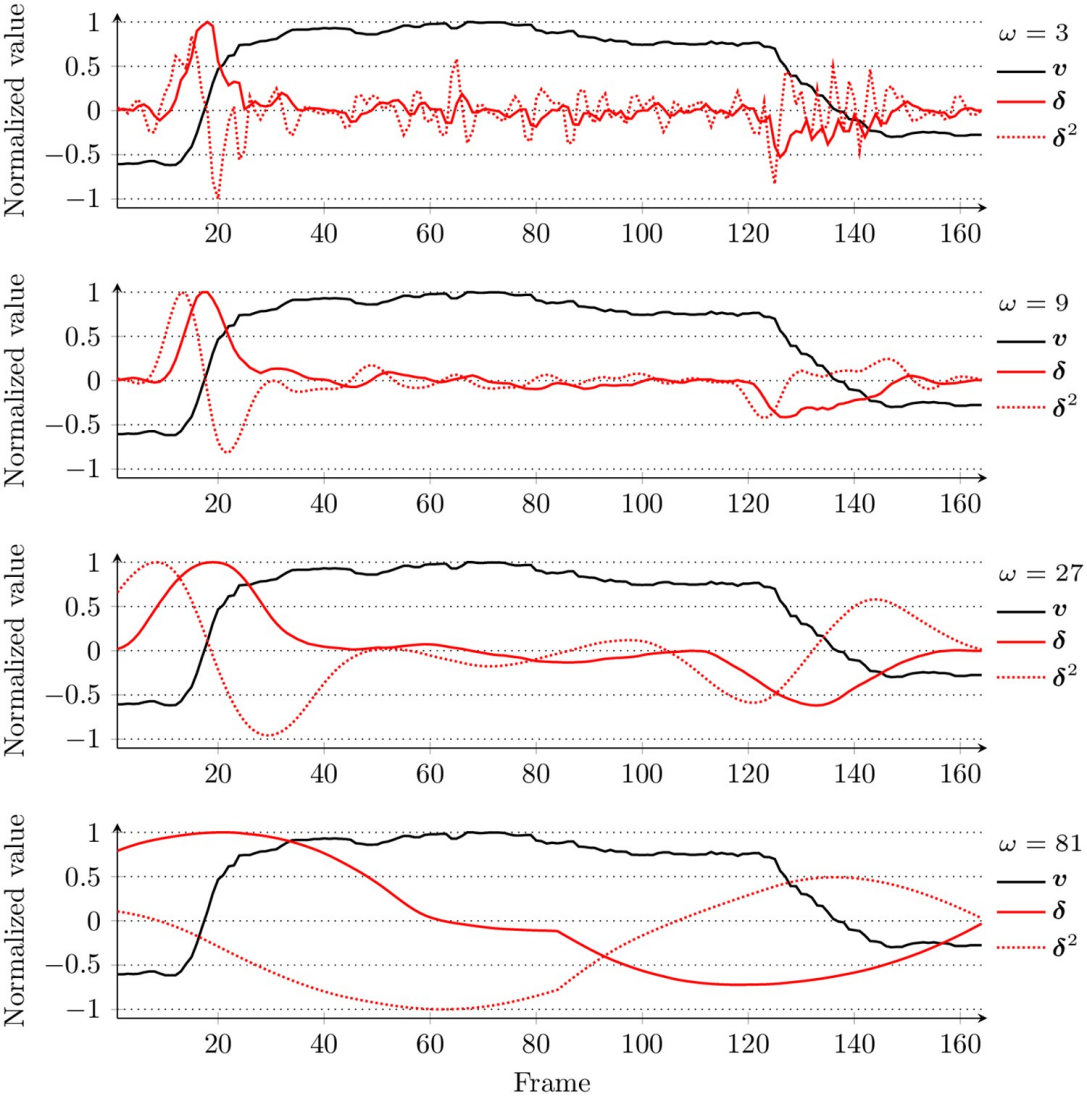
An example (file 020_spontaneous_smile_2 from UvA-NEMO) of a smile intensity series (*v*, captured at 50 fps) along with extracted first-order (*δ*) and second-order (*δ*^2^) dynamics, extracted using different window sizes (*ω*). For visualization purposes, values of all the signals were normalized.

For every *i*-th intensity in the sequence (*v*_*i*_), we compute *δ*_*i*_, hence the ***v*** signal is transformed into ***δ*** that represents its first-order dynamics. We replicate the boundary values when processing the initial or final values. By applying different window lengths (hence obtaining a variety of ***δ*** series), we determine the dynamics at different scales. We also extract the second-order dynamics by processing ***δ*** once again, which produces the ***δ***^2^ signal. In addition to that, the slope value can be adjusted based on the regression coefficient—the *r*-adjusted values are obtained as ${\hat{\delta }}_{i}=\delta_{i}\left|r_{i}\right|$. In Fig 2, we present an example of ***δ*** and ***δ***^2^ signals obtained from the smile intensity series using windows of different lengths (the length must be an odd number, and we demonstrate the signals for subsequent powers of 3). It can be seen that for *ω* = 3, the noise influences the ***δ*** and ***δ***^2^ signals, but for longer windows (e.g., *ω* = 9 and *ω* = 27, corresponding to 160 ms and 520 ms), the very smile dynamics are well captured. On the other hand, a larger scale (such as *ω* = 81) may not be suitable for highlighting the dynamics of smiles that are a few seconds long. Therefore, we decided to focus on the range 9 ≤ *ω* ≤ 27.

### Detecting smile phases

We estimate the smile intensity for every frame using an SVM trained to classify AU feature vectors as presenting a smile or not—the intensity of a smile is determined based on the distance from the hyperplane that separates the opposite classes (i.e., *smile* and *non-smile*), whose position is found during the SVM training. Although such an approach was reported to be not as effective as when multiclass or regression models are used [51], the latter require training sets with continuous smile intensity labels (or multiple intensity classes) that are difficult to acquire and prone to annotation errors. Contrary to that, the binary ground-truth labels are less problematic to obtain, and we have found an SVM trained with them sufficient to analyze the dynamics of the smile intensity signal and to detect the smile phases. Due to the large sizes of the training sets and their imbalance (the *smile* frames being the majority class), we employed our training set selection algorithm [63] to train an SVM.

**Algorithm 1** An algorithm to determine the subsequent smile phases (onset: *t*_on_ to *t*_ap_, apex: *t*_ap_ to *t*_off_, and offset: *t*_off_ to *t*_end_).

1: Input signals: ***v***, ***δ***, ***δ***^2^ of length *T*;

2: Output: ***P***;          ⊳*P_i_* {onset, apex, offset, none}

3: *t*_0_ ← 1;          ⊳Indicates a current smile starting point

4: **repeat**

5:  *t*_on_ ← FindFirst(***δ***, *δ* > 0, *t*_0_);

6:  *v*_ref_ ← *v*_on_;          ⊳*v*_on_ indicates a value of ***v*** at *t*_on_ ($v_{i}\equiv v_{t_{i}}$)

7:  *t*_*c*_ ← 1;          ⊳*v*_on_ Indicates a current position of the search

8:  *v*^max^ ← *v*_*c*_;

9:  **repeat**      ⊳A loop to determine the final smile intensity descent

10:   **if** *v*_*c*_ > *v*^max^ **then**;

11:    *v*^max^ ← *v*_*c*_;

12:    *v*_ref_ ← (*v*_on_ *v*^max^)/2;

13:   **end if**

**Fig 3:**
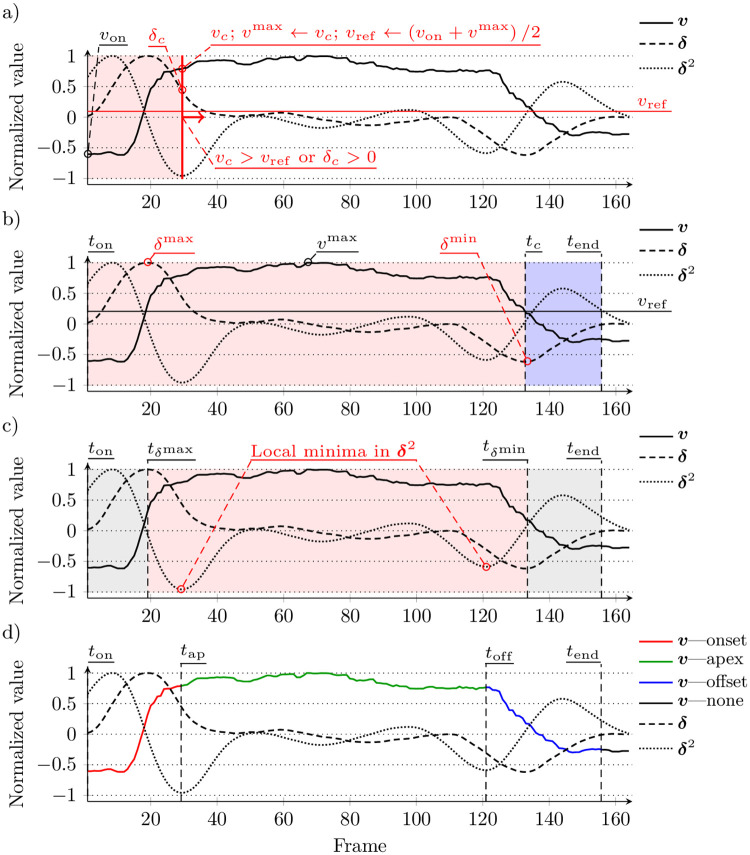
Illustrative example of detecting smile phases: a) search for the final smile descent (*t*_*c*_ to *t*_end_), b) the *δ* extrema used to determine the preliminary apex limits, c) the apex boundaries fine-tuned relying on *δ*^2^ local minima, d) final outcome. The smile intensities were extracted from the file 020_spontaneous_smile_2 from the UvA-NEMO database.

14:   *t*_*c*_ ← *t*_*c*_ + 1;

15:  **until** (*v*_c_ > *v*_ref_ or *δ*_*c*_ > 0) and *t*_*c*_ < *L*;

16:  *t*_end_ FindFirst(***δ***, *δ* > 0, *t*_*c*_);

17:  *t*_*δ*^max^_ ← FindMax(***δ***, ❮*t*_on_, *v*_end_❯);

18:  *t*_*δ*^min^_ ← FindMin(***δ***,❮*t*_on_, *v*_end_❯);

19:  *t*_ap_ ← FindFirstLocalMinimum(***δ***^2^, ❮_*δ*^max^, *t*_*δ*^min^__〉);

20:  *t*_off_ ← FindLastLocalMinimum(***δ***^2^, ❮_*δ*^max^_, *t*_*δ*^min^_〉);

21:  *t*_ap_, *t*_off_ ← ValidateApex(*t*_*δ*^max^_, *t*_*δ*^min^_, *t*_ap_, *t*_off_);

22:  *t*_0_ ← *t*_end_;

23:  SetPhases(***P***, *t*_on_, *t*_ap_, *t*_off_, *t*_end_);

24: **untill** *t*_0_ < *L*;

We determine a vector of the smile phases (*P*) based on the relative changes observed in the estimated smile intensity signal ***v***, as well as in its first-order and second-order dynamics (***δ*** and ***δ***^2^). To obtain these signals, we use a window of *ω* = 27 (for 50 fps). In contrast to the existing approaches, we take no assumptions on the number of phases in a presented sequence. Algorithm 1 presents the procedure for detecting the smile phases and the process is illustrated in Fig 3. The search of a new smile starts when *δ* > 0 (line 5) and it is composed of three major steps, whose goal is to: (*i*) determine the temporal extent of the smile event, (*ii*) find the approximate limits of the apex phase, and (*iii*) fine tune the apex boundaries.

First, the signal is scanned to find the final descent of the smile intensity (lines 9–15). During that step, visualized in Fig 3a, the signal is scanned as long, as the current smile intensity value is over a continuously updated reference value (*v*_c_ > *v*_ref_). To avoid stopping in case of incidental low *v* (e.g., resulting from noise), it is required that *δ* < 0 to finish the search. This determines the *t*_*c*_ timestamp, after which the next positive value of *δ* is considered as the end of the current smile event (line 16). This determines the boundaries of the current smile (*t*_on_ to *t*_end_).

During the second step (Fig 3b), the ***δ*** signal is analyzed within the detected range ❮*t*_on_;*t*_end_❯. We assume that the fastest increase (*δ*^max^) happens during the onset, while the fastest decrease (*δ*^min^) during the offset phase (lines 17 and 18). This sets the initial limits of the apex phase (we expect it to start after *t*_*δ*^max^_ and finish before *t*_*δ*^min^_).

The third step consists in inspecting the ***v***’s second-order dynamics to find the maximum convexity of the smile intensity which would indicate the apex phase’s bounds (Fig 3c). For this purpose, we scan ***δ***^2^ for the first (line 19) and last (line 20) local minimum in the range _*δ*^max^, *t*_*δ*^min^_〉_. If the local minima are not found, then we use the initial limits (*t*_*δ*^max^_ and *t*_*δ*^min^_) determined during the second step (line 21). Finally, we validate the determined limits (we also check whether the smile lasts at least 1 second) to approve the detected phases (line 23 and Fig 2d).

### Classifying the smiles based on facial actions dynamics

Every detected smile is classified based on the features extracted from AU sequences within each individual smile phase, as well as from the entire smile cycle (i.e., from *t*_on_ till *t*_end_). We extract two types of features, namely *AU-wise* features, derived independently from each individual AU, and *cross-AU* features that capture the mutual relations between the AUs. The AU-wise features embrace the amplitude of the signal (*v*_*a*_ = *v*^max^ − *v*^min^), the average and maximum values ($\bar{v}$ and *v*^max^), as well as the average and maximum values of the first-order dynamics (*δ*^max^ and $\bar{\delta }$), extracted at *ω* = 9 and *ω* = 27 (at 50 fps). Hence, for 17 AUs we obtain 51 features (*v*_*a*_, $\bar{v}$ and *v*^max^ for each AU) plus 34 features (*δ*^max^ and $\bar{\delta }$) per every value of *ω* (119 features for *ω* ∈ {9, 27}).

The purpose of the cross-AU features is to retrieve the dependencies between the dynamics of individual AU signals. For every pair of AUs, we compute the dynamics difference signal:

$$\begin{array}{c} \delta_{\Delta }(\text{AU}x,\text{AU}y)=|\delta (\text{AU}x)-\delta (\text{AU}y)| \end{array}$$ (3)

to take minimum ($\delta_{\Delta }^{\text{min}}$) and maximum ($\delta_{\Delta }^{\text{max}}$) values as features. In addition to that, we locate the minimum and maximum for the *r*-adjusted dynamics, and for each pair of AUs, we consider their distance in the temporal domain ($\Delta t_{{\hat{\delta }}^{\text{max}}}$ and $\Delta t_{{\hat{\delta }}^{\text{min}}}$). In this way, we retrieve information on whether the maximum linear increase (or decrease) in two AU signals are close to each other. For a single value of *ω*, we obtain 544 cross-AU features.

**Fig 4:**
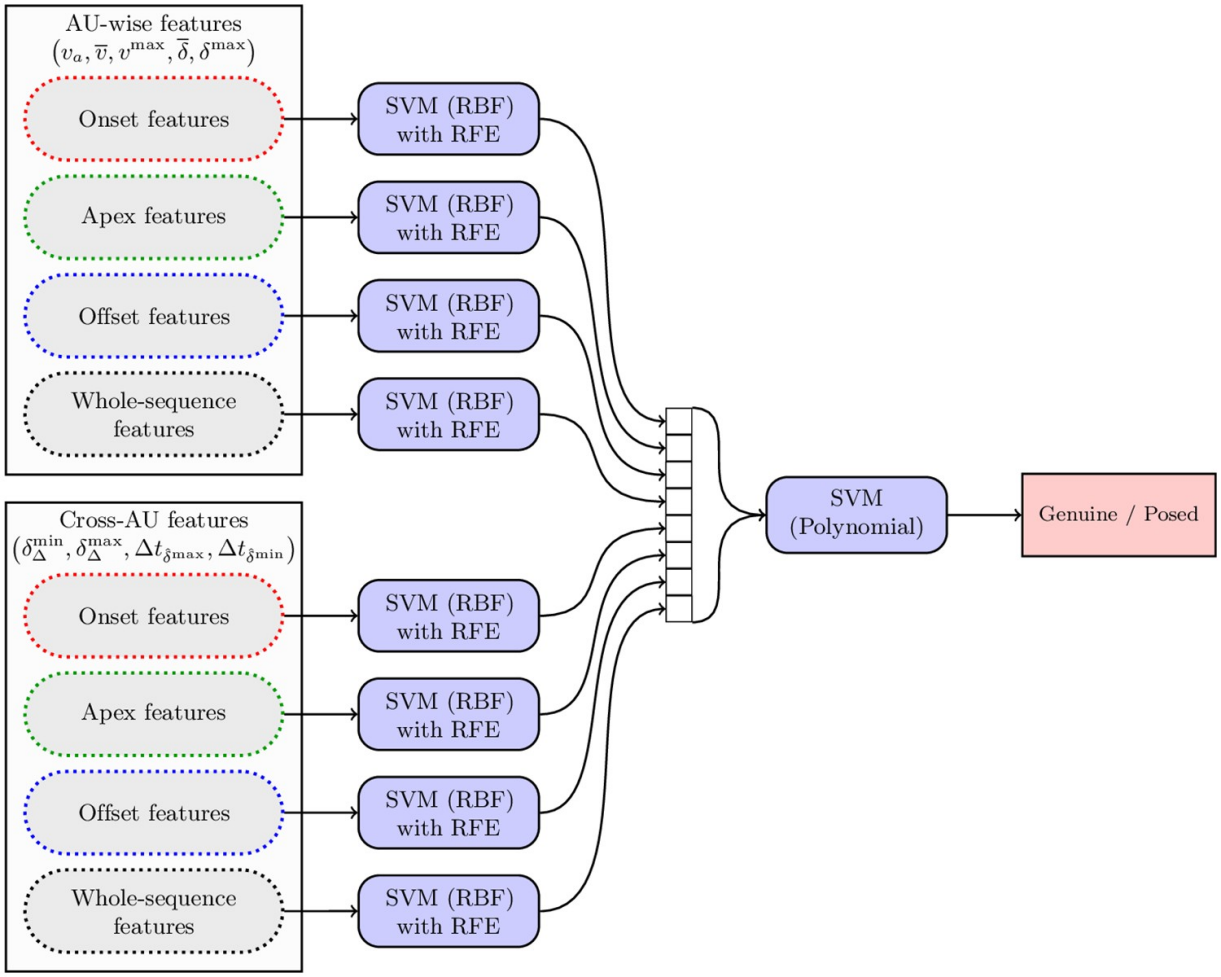
A double-level classification scheme to classify the AU-wise and cross-AU features extracted from the detected smile phases, as well as from the entire sequence.

The aforementioned two types of features are extracted from four different time ranges that reflect the smile phases, hence we obtain eight feature vectors, as presented in Fig 4. Each feature is subject to standardization based on the training set, and the obtained feature vector is classified using an SVM with a Radial Basis Function (RBF) kernel. During training, the features are selected with Recursive Feature Elimination (RFE) [64] to simplify the model. We assess the importance of each individual feature by excluding it from the feature set to observe the performance of the model trained without that feature. The least important features are recursively eliminated (we allow for eliminating multiple features at a time), as long as the classification performance, measured for the validation set, does not decrease. The validation set is a part of the training set (not to be confused with the test set which remains unseen during that procedure). Finally, we treat these first-level SVMs as an ensemble—the SVM responses (i.e., the distances from separating hyperplanes) are treated as the elements of a second-level feature vector which is classified using an SVM with a polynomial kernel. This produces the final decision on whether the considered smile is spontaneous or posed.

## Results and discussion

### Experimental setup

We evaluate the proposed algorithm using two benchmarks created for assessing smile genuineness recognition: UvA-NEMO database [65] which contains 1240 video sequences of posed and spontaneous smiles (643 and 597 sequences, respectively, involving 400 subjects) with a resolution of 1920 × 1080 pixels, captured at 50 fps, and the BBC database (available at http://www.bbc.co.uk/science/humanbody/mind/surveys/smiles) with 20 video sequences (10 posed and 10 spontaneous smiles of 20 different subjects), captured at 25 fps, with a resolution of 314 × 286 pixels. For UvA-NEMO, we followed the official evaluation protocol published by the database authors (the UvA-NEMO database alongside all the metadata and division into the folds are available at https://www.uva-nemo.org/index.html) which is based on 10-fold cross validation—SVMs are trained with 9 folds, and the performance is tested for the remaining fold unseen during training (the subjects whose images are in the test set do not appear in the training set). The process is repeated for every fold, and the scores obtained for the test sets are averaged over all the folds. For the BBC database, we report the scores using 10-fold cross validation.

For detecting AUs, we exploit the OpenFace library which implements the SVM-HOG method [46]. Our algorithms for capturing the dynamics of facial expressions, detecting the smile phases, followed by extraction and classification of the features, were implemented in the C++ language with the use of the libsvm library. The SVM hyper-parameters were determined based on a grid search, performed for every fold. The validation sets used to evaluate the model during the grid search and feature selection procedures were extracted from the training set, hence the test set remained unseen during training. To compare the proposed features with alternative approaches, we have also implemented the feature extraction in the Facial Landmark Analysis (FLA) method by Dibeklioğlu et al. [4], and we classified them with SVM. We ran our experiments on a computer equipped with an Intel Core i7-3740QM 2.7 GHz (32 GB RAM) processor. Processing a sequence composed of 100 frames consumes 3 ms to identify the smile phases, 6 ms to extract and classify the AU-wise features, and 153 ms to extract and classify the cross-AU features. Overall, this allows for real-time analysis.

Experimental validation is composed of three major parts that are presented and discussed later in this section: (*i*) evaluation of smile phase detection, (*ii*) analysis of the proposed AUDA method, (*iii*) comparison with the state of the art. The performance of recognizing smile genuineness is evaluated based on the classification accuracy (the percentage of correctly classified samples) as well as with the area under the receiver operating characteristic curve (AUC).

### Evaluation of smile phase detection

As there are no ground-truth data available on when the particular smile phases start and finish, the accuracy of the proposed smile phase detection algorithm cannot be determined directly by comparing the outcome against the reference. Therefore, we evaluated the algorithm qualitatively, by inspecting the obtained outcome, and quantitatively to assess: (*i*) the algorithm’s behavior for sequences presenting multiple smiles and (*ii*) its robustness against the noise injected into the smile intensity signal.

**Fig 5:**
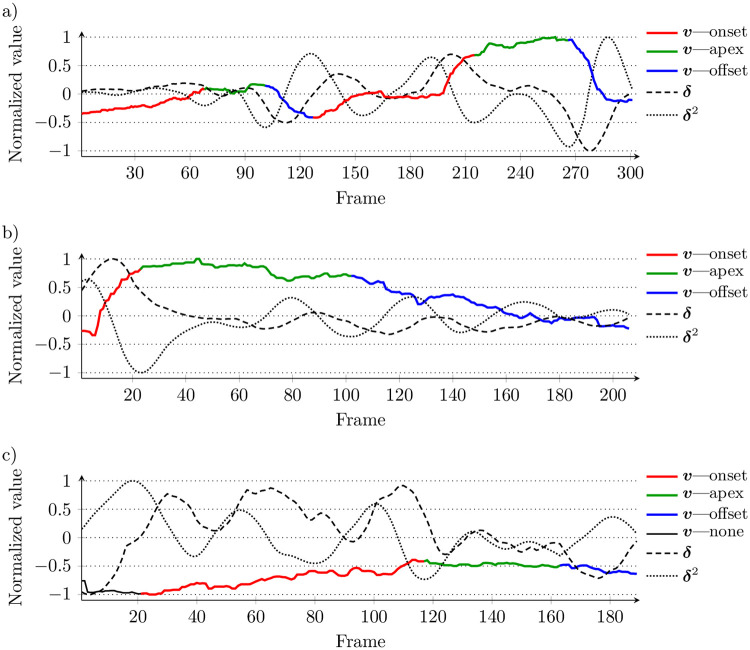
Examples of detected smile phases derived from UvA-NEMO database (files 002_spontaneous_smile_2, 011_deliberate_smile_1, and 015_spontaneous_smile_4). All of these sequences were later correctly classified with the proposed AUDA algorithm.

In the example presented earlier in Fig 3, the smile phases were clearly visible and they were correctly identified. Fig 5 demonstrates three examples of non-obvious cases. In Fig 5a, the sequence contains two cycles of the smile intensity—as it can be seen from the plots, they have been correctly identified and split into three phases. Fig 5b shows a case with rather smooth transition between the apex and offset phases and in Fig 5c, the smile intensity remains low across the whole sequence—in fact, the smile is not detected with the binary frame-wise classifier (which is wrong, looking at the corresponding frames presented over the plot), but the smile and the smile phases are identified correctly by analysing the intensity dynamics.

We expect the smile phases to be identified regardless of the length of the presented sequence and the number of smile events. In order to verify that, we combined all the original single-smile sequences from UvA-NEMO into a single long sequence. We treat the smile phases detected from the original sequences as a reference, and we compare them against the phases identified from the long combined sequence. In Table 1, we report the confusion matrices for spontaneous and posed smiles that show the differences between detecting the smile phases in these two scenarios. It can be seen that the frames classified as belonging to the apex phase from the original sequences are mostly classified as apex from the long sequence (97.9% and 96.3% for posed and spontaneous smiles, respectively), and the differences are mainly in the lengths of the onset and offset phases. It is quite common that given a broader context in the long sequences, the offset phase is moved forward (making the apex phase longer). Overall, despite some discrepancies, the phase detection was stable for multiple smile events in a sequence, making it suitable for continuous analysis—over 90% of the frames were assigned the same phase in both scenarios.

**Table 1:** Confusion matrices showing the differences between smile phase detection performed for original sequences from the UvA-NEMO database (which contain a single smile per sequence) vs. a long combined sequence composed of the single-smile ones. Bold values indicate the numbers of frames whose phase match in both approaches.

Single-smile ↓		Long combined sequence				
		None	Onset	Apex	Offset	Matched
Posed	None	1885	712	112	824	—
	Onset	728	**17336**	519	1219	87.5%
	Apex	478	235	**52012**	411	97.9%
	Offset	743	1055	2600	**18233**	80.6%
	Matched	—	89.6%	94.2%	88.1%	93.5%
Spontaneous	None	3010	861	317	936	—
	Onset	1343	**21612**	2148	1765	80.4%
	Apex	1025	917	**83401**	1271	96.3%
	Offset	1773	984	5338	**20506**	71.7%
	Matched	—	88.7%	91.4%	83.8%	91.0%

**Fig 6:**
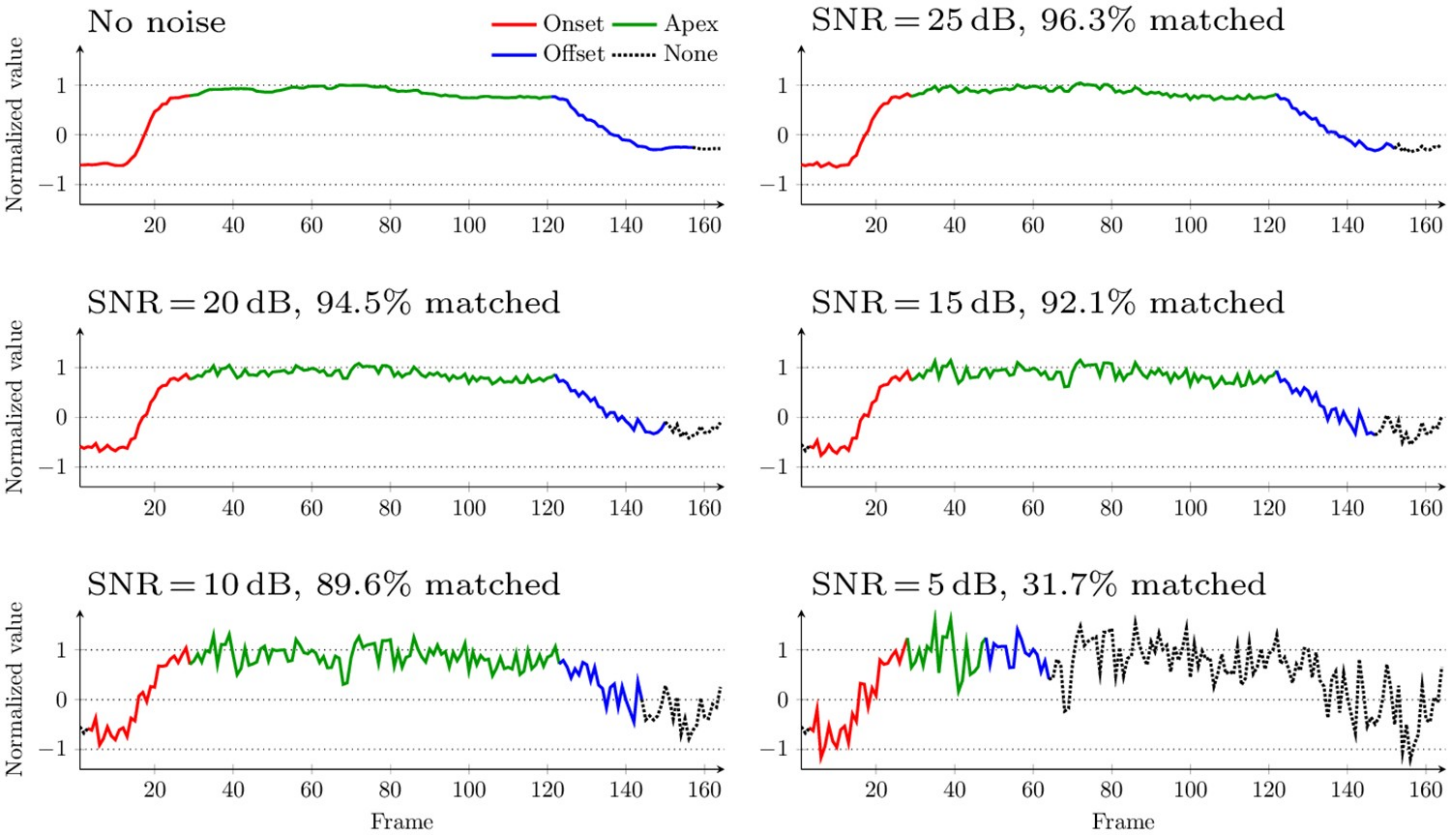
Smile intensity signal contaminated with different amounts of Gaussian noise. In this case (file 020_spontaneous_smile_2 from UvA-NEMO), smile phase detection works correctly for SNR of at least 10 dB.

In other works on recognizing the smile genuineness [4, 5], it is assumed that the onset phase is the longest continuous increase of the smile intensity (measured as the distance between the lip corners), making it quite vulnerable to the noisy values (e.g., resulting from imprecise detection of the landmarks). Our algorithm was designed to be robust against the noisy data, hence we investigated its behavior in the presence of the Gaussian noise. We have contaminated the smile intensity signals with different levels of the Gaussian noise to obtain signal-to-noise ratio (SNR) of 5, 10, 15, 20, and 25 dB, and we detected the smile phases from these noisy data. In Fig 6, we show an example of the smile intensity signal with different levels of the noise, and in Table 2 we report the percentage of frames whose identified phase was not affected by the noise. It can be seen that phase detection is more vulnerable for the spontaneous smiles (in general, the intensity signals are less smooth here than for the posed smiles), however in both cases the detection remains stable for SNR of at least 20 dB.

**Table 2:** Sensitivity of the phase detection algorithm to the additive Gaussian noise for sequences from the UvA-NEMO database showing posed and spontaneous smiles. The scores show the percentage of the frames whose identified phase was not affected by the noise.

Signal-to-noise ratio	Matched frames [%]	
	Posed	Spontaneous
25 dB	97.39	96.36
20 dB	95.90	92.30
15 dB	92.18	83.18
10 dB	81.61	64.23
5 dB	52.80	41.68

### Analysis of the proposed smile genuineness recognition

At first, we investigated the classification performance for the features extracted from individual AUs (for the AU-wise features) and pairs of AUs (for the cross-AU features). In Table 3, we report the scores obtained for the features extracted from each individual AU within the whole detected sequence (i.e., between *t*_on_ and *t*_end_) and from each smile phase. In addition to that, we combine these four classifiers using the SVM with a polynomial kernel. In general, the accuracy is similar for the features extracted from the whole sequence and for those derived from the onset phase, and it is lower for those extracted from apex and offset. Importantly, for all AUs, the SVM ensemble renders a higher classification accuracy than the phase-wise SVMs which exposes the importance of identifying the smile phases. It can be seen that the most discriminative are the dynamics of AU12 (lip corner puller), AU6 (cheek raiser), and AU10 (upper lip raiser), followed by AU25 (lips part), AU14 (dimpler), and AU5 (upper lid raiser). Interestingly, the dynamics of each individual AU, including AU45 (blinking), allow for obtaining the classification accuracy of over 65%.

**Table 3:** Classification accuracy (in %) obtained for the UvA-NEMO database using AU-wise features extracted from individual AUs. The features were extracted from each smile phase (onset, apex and offset), as well as from the whole sequence. The “combined” column shows the scores obtained using an ensemble of four AU-wise SVM classifiers (as shown in Fig 4). The darker the background, the higher the accuracy is.

AU	Whole	Onset	Apex	Offset	Combined
1	64.55	65.85	56.62	54.7	68.51
2	62	63.06	58.09	57.66	67.29
4	62.94	65.8	60.92	55.4	67.94
5	66.87	66.14	62.06	55.98	70.46
6	75.56	75.89	64.33	55.95	76.38
7	65.19	67.82	64.29	57.99	68.64
9	62.5	67.07	59.85	56.03	68.37
1	72.36	71.67	61.07	56.16	74.64
12	77.99	74.27	68.05	64.58	78.95
14	67.47	70.25	64.73	60.59	71.41
15	65.52	65.25	59.17	55.56	68.98
17	61.19	64.55	59.77	55.54	66.2
2	63.6	64.9	59.64	55.26	67.78
23	63.93	64.42	60.15	60.24	66.79
25	68.12	69.63	59.67	56.74	72.9
26	62.15	64.33	59.05	57.46	65.89
45	63.27	63.96	58.22	53.38	66.55

Table 4 shows the scores obtained using the cross-AU features extracted from the individual pairs of AUs. Here, we report the final classification accuracy obtained with the ensemble of four SVMs trained with the feature vectors extracted from the individual smile phases. It can be observed that the best scores (over 70%) are obtained relying on the pairs that include AU12 (lip corner puller) and AU6 (cheek raiser), especially when coupled with AUs that code the behavior of lips and mouth (including AU15—lip corner depressor, AU17—chin raiser, AU20—lip stretcher and AU26—jaw drop). However, the effectiveness of the pair AU6-AU12 is relatively low—as it was noted in [66], these AUs are correlated with each other, and possibly this correlation does not differ significantly between spontaneous and posed smiles. It is worth noting that quite high scores are obtained for AU2 (outer brow raiser) coupled with AU6 and AU12, as well as with AU10 (upper lip raiser) and AU7 (lid tightener)—this confirms some of the earlier findings [4, 6, 59] on the importance of the correlation between the mouth and eye regions.

**Table 4:** Classification accuracy (in %) obtained for the UvA-NEMO database using cross-AU features extracted from particular pairs of AU signals. The scores were obtained using an ensemble of four cross-AU SVM classifiers (as shown in Fig 4). The darker the background, the higher the accuracy is.

AU	1	2	4	5	6	7	9	10	12	14	15	17	20	23	25	26
2	57.54															
4	56.46	62.76														
5	61.01	61.19	61.46													
6	71.02	74.47	73.96	72.88												
7	64.81	72.15	66.56	64.81	61.95											
9	63.5	63.01	63.78	59.79	72.39	60.79										
1	71.03	73.1	65.98	70.65	62.04	61.92	63.53									
12	75.35	78.47	75.36	76.35	69.82	70.42	74.36	70.68								
14	62.87	68.85	62.35	66.58	72.39	62.47	60.4	67.28	75							
15	61.68	63.89	62.34	63.69	74.27	66.18	63.36	70.86	78.56	69.29						
17	58.93	58.88	59.55	58.77	73.44	68.9	61.96	69.43	77.86	68.27	61.47					
20	61.11	61.24	59.68	59.15	74.89	67.58	60.84	69.25	74.98	62.89	60.75	61.17				
23	57.74	58.54	59.58	58.85	72.73	69.83	60.23	70.21	75.86	63.19	59.43	58.87	57.72			
25	64.43	66.1	65.59	64.86	67.8	64.23	65.55	63.24	64.1	64.75	66.37	68.29	65.38	70.96		
26	62.07	64.85	60.34	59.72	72.14	63.42	61.5	66.44	78.06	64.65	63.99	63.3	63.3	62.56	65.86	
45	62.01	55.57	57.23	60.45	70.77	63.54	60.17	70.22	71.98	66.06	63.94	62.56	58.66	60.77	66.81	63.54

In Table 5, we report the classification accuracy and AUC for several variants of the proposed AUDA method, including the use of exclusively the AU-wise features (for all AUs) and the cross-AU features (for all of the AU pairs). Also, we investigate the scores for the dynamics extracted using different sets of the window lengths (*ω*). As it was discussed earlier in the paper and demonstrated in Fig 2, the sensible values of *ω* are between 9 and 27 at 50 fps, which corresponds to the windows of 160 ms and 520 ms. During the experiments, we have sampled that range more densely, adding the values of *ω* = 15 (280 ms) and *ω* = 21 (400 ms). As the standard deviations across the folds are considerable (compared with the differences between the variants), we employed the two-tailed Wilcoxon test to verify the hypothesis that the variants do *not* differ between each other. For the accuracy and AUC, we boldfaced the highest score, and the variants for which the hypothesis has been rejected at *p* < 0.05 were underlined. The best results were obtained using all the features extracted from two (*ω* ∈ {9, 27}) and four windows (*ω* ∈ {9, 15, 21, 27}) without any statistically significant difference between these variants. As they are significantly different from the single-window variants (for *ω* = 9 and *ω* = 27), we decided to use the two-window variant as our baseline. It is also clear from the table that using all the features delivers better scores than relying exclusively on cross-AU and AU-wise features, which justifies exploiting both types of them. It may also be noted that including all the AU pairs renders higher accuracy (81.23%) than the score obtained with a single pair in Table 4 (i.e., 78.56% for AU12-AU15). Similarly, the best score obtained for a single AU in Table 3 (i.e., 78.95% for AU12) is lower than using all the AUs (82.25%).

**Table 5:** Scores (classification accuracy and AUC) obtained for UvA-NEMO database using different variants of our AUDA method. The best score in each column is marked as bold and the scores that are not significantly different from the best (in the statistical sense) are underlined.

Features	*ω* (at 50 fps)	Accuracy [%]	AUC
All	9	83.28 ± 3.12	0.8953 ± 0.0320
All	27	83.59 ± 2.97	0.8929 ± 0.0322
All	9, 27	**84.56 ± 3.29**	0.9029 ± 0.0341
All	9, 15, 21, 27	84.39 ± 3.38	**0.9041 ± 0.0339**
AU-wise	9	81.05 ± 3.31	0.8703 ± 0.0300
AU-wise	27	80.61 ± 3.20	0.8605 ± 0.0359
AU-wise	9, 27	82.25 ± 3.49	0.8784 ± 0.0340
AU-wise	9, 15, 21, 27	82.25 ± 3.10	0.8829 ± 0.0350
Cross-AU	9	80.36 ± 4.09	0.8607 ± 0.0394
Cross-AU	27	78.96 ± 3.70	0.8589 ± 0.0396
Cross-AU	9, 27	81.23 ± 3.30	0.8772 ± 0.0368
Cross-AU	9, 15, 21, 27	81.38 ± 3.99	0.8744 ± 0.0376

For the selected baseline variant, we performed the RFE-based feature selection. In Table 6, we report the performance of the first-level SVMs, as well as of the final classification ensemble, trained without and with feature selection. Using the selected subset of features, the scores are slightly worse than when SVMs are trained from all the features extracted from the whole sequence, as well as for the cross-AU features extracted from the apex phase. The performance of the remaining first-level SVMs and that of the final ensemble is better after applying feature selection. It is worth noting that when using all AU-wise features extracted from the onset, apex and offset phases, the classification accuracy is lower than for SVMs trained based on individual AUs (Table 3). For example, SVMs trained from AU6, AU10, AU12, AU14, and AU25 onset features are better than using all AUs. After feature selection, these scores are higher than relying on any single AU.

**Table 6:** Scores obtained for the UvA-NEMO database using all the features and those selected using recursive feature elimination.

Features ↓		All features (no selection)		RFE-selected features	
		Accuracy [%]	AUC	Accuracy [%]	AUC
AU-wise	Whole	82.08 ± 3.55	0.8764 ± 0.0356	81.74 ± 3.58	0.8746 ± 0.0309
	Onset	69.40 ± 4.39	0.7236 ± 0.0466	76.97 ± 3.98	0.8154 ± 0.0475
	Apex	58.78 ± 3.55	0.5589 ± 0.0448	74.02 ± 2.85	0.7736 ± 0.0378
	Offset	63.91 ± 3.81	0.6464 ± 0.0564	72.05 ± 4.32	0.7588 ± 0.0436
Cross-AU	Whole	80.11 ± 2.75	0.8629 ± 0.0393	79.07 ± 3.26	0.8465 ± 0.0387
	Onset	71.40 ± 3.73	0.7339 ± 0.0405	74.87 ± 4.20	0.7918 ± 0.0528
	Apex	78.00 ± 4.62	0.8320 ± 0.0413	76.73 ± 4.11	0.8215 ± 0.0364
	Offset	72.05 ± 4.90	0.7504 ± 0.0487	74.76 ± 1.90	0.7968 ± 0.0323
Final ensemble		84.56 ± 3.29	0.9029 ± 0.0341	85.11 ± 3.46	0.9073 ± 0.0314

In Table 7, we report the ratios of the selected features grouped by the action unit they originate from (for the cross-AU features, each feature originates from two AUs). Similarly, in Table 8, the ratios are categorized by the particular dynamics extracted from all AUs. It can be observed that there is substantial information redundancy among the features and in most cases over half of them can be rejected without affecting the final classification performance (more for the cross-AU features). From Table 7, it can be seen that the features related with AU6 and AU12 (and AU25 for AU-wise) were more often picked than those related with other AUs. This is coherent with the observations made for the classifiers based on single AUs (Table 3) and their pairs (Table 4), discussed earlier in this section. However, some AUs (e.g., AU10) that rendered high classification scores when treated individually, were not that often selected with RFE. Importantly, even though the features related with some AUs were selected more frequently, none of AUs nor feature types were entirely eliminated which confirms that all of the proposed features that capture the AUs’ dynamics are relevant for discriminating between posed and spontaneous smiles.

**Table 7:** Ratio of selected features (in %) associated with individual action units for the AU-wise and cross-AU features extracted from different smile phases, averaged across all folds for the UvA-NEMO database. There are 7 and 128 features associated with each AU for the AU-wise and cross-AU features, respectively. The darker the background, the higher the ratio is.

AU ↓	AU-wise					Cross-AU				
	Whole	Onset	Apex	Offset	Avg.	Whole	Onset	Apex	Offset	Avg.
1	40.00	21.43	51.43	27.14	35.00	21.80	15.16	27.34	18.67	20.74
2	38.57	15.71	51.43	27.14	33.21	22.89	12.19	28.36	20.47	20.98
4	34.29	11.43	47.14	21.43	28.57	23.67	15.16	31.41	19.06	22.32
5	48.57	32.86	54.29	40.00	43.93	21.25	15.00	29.61	23.05	22.23
6	55.71	30.00	70.00	37.14	48.21	27.11	17.73	33.44	20.47	24.69
7	22.86	28.57	50.00	41.43	35.71	20.39	15.55	26.48	19.22	20.41
9	42.86	12.86	41.43	38.57	33.93	21.72	15.31	28.59	18.44	21.02
10	34.29	21.43	51.43	38.57	36.43	21.80	17.81	31.80	17.89	22.32
12	65.71	47.14	75.71	64.29	63.21	27.66	16.80	35.31	22.58	25.59
14	45.71	20.00	50.00	31.43	36.79	22.66	17.50	28.36	20.23	22.19
15	40.00	32.86	38.57	32.86	36.07	21.41	16.09	31.09	20.70	22.32
17	37.14	17.14	58.57	27.14	35.00	21.09	16.25	30.08	17.89	21.33
20	31.43	12.86	45.71	34.29	31.07	25.23	15.55	27.89	21.33	22.50
23	40.00	30.00	64.29	40.00	43.57	22.73	16.64	32.66	18.83	22.71
25	61.43	41.43	60.00	51.43	53.57	24.77	15.23	31.88	15.70	21.89
26	35.71	38.57	50.00	47.14	42.86	23.44	18.28	29.14	21.17	23.01
45	25.71	30.00	45.71	38.57	35.00	24.30	19.06	28.28	18.36	22.50
All	41.18	26.13	53.28	37.56	39.54	23.17	16.19	30.10	19.65	22.28

**Table 8:** Ratio of selected features (in %) for each feature type extracted from different smile phases, averaged across all folds for the UvA-NEMO database. There are 17 and 136 features of each kind extracted from individual AUs (for the AU-wise features) and their pairs (for the cross-AU features), respectively. The darker the background, the higher the ratio is.

Feature ↓		*ω*	Whole	Onset	Apex	Offset	Avg.
AU-wise	*v*_*a*_	—	57.65	25.88	49.41	33.53	41.62
	$\bar{v}$	—	31.18	25.29	47.06	40.00	35.88
	*v*^max^	—	35.29	22.35	51.18	27.65	34.12
	$\bar{\delta }$	9	35.88	25.88	49.41	44.12	38.82
		27	37.06	24.71	58.24	41.18	40.29
	*δ*^max^	9	45.29	34.12	55.29	35.29	42.50
		27	45.88	24.71	62.35	41.18	43.53
	All		41.18	26.13	53.28	37.56	39.54
Cross-AU	$\delta_{\Delta }^{\text{min}}$	9	26.25	21.18	32.79	20.15	25.09
		27	25.66	17.65	31.10	24.49	24.72
	$\delta_{\Delta }^{\text{max}}$	9	28.46	18.60	30.22	19.78	24.26
		27	20.29	12.50	33.82	23.09	22.43
	$\Delta t_{{\hat{\delta }}^{\text{min}}}$	9	21.54	15.29	30.22	18.97	21.51
		27	19.85	14.41	27.57	19.12	20.24
	$\Delta t_{{\hat{\delta }}^{\text{max}}}$	9	20.00	16.62	25.15	14.04	18.95
		27	23.31	13.31	29.93	17.57	21.03
	All		23.17	16.19	30.10	19.65	22.28

### Comparison with the state of the art

In Tables 9 and 10, we compare the obtained classification accuracy and AUC with the state-of-the-art techniques for the UvA-NEMO and BBC databases, respectively. The best results were reported for the DLM method [5] applied to classify the disCLBP-TOP features extracted from spatial-temporal blocks. The authors in [5] stated that their disCLBP-TOP features exploit information concerned with facial appearance which conveys the age of a person. Therefore, they compare their DLM method against the FLA+Age variant from [4] that benefits from the age-based stratification. As the AUDA features are extracted from the AU signals, they do not capture the facial appearance. Also, it is worth noting that most of the existing methods (including DLM, disCLBP-TOP, and FLA) assume that every presented sequence contains a single smile event, which simplifies the process of identifying the subsequent smile phases. Our AUDA method is not restricted in this way, allowing for processing continuous smile sequences. The original FLA method [4] is based on the geometric features, from which the most discriminative ones are selected using min-redundancy max-relevance algorithm before final classification with an SVM. We also report the scores obtained with our implementation of the FLA method (without the feature extraction step, termed FLA-all), which we combined with the classifiers based on the AUDA features (an SVM trained from FLA-all is included as the ninth classifier in our ensemble). For UvA-NEMO, such a combination (AUDA+FLA-all variant) renders better results than both AUDA and FLA-all, and the difference is statistically significant according to the Wilcoxon test (at *p* < 0.05). For BBC, the FLA-all features occurred to be less effective (for 10-fold split they do not improve the results when combined with our method).

**Table 9:** Scores (classification accuracy and area under the ROC curve) obtained for the UvA-NEMO database using different methods. For the scores directly quoted from other papers, an appropriate reference is provided.

Method	Accuracy [%]	AUC
SOAD [53]	77.26	—
CLBP-TOP [57]	73.06	—
CLBP-TOP+ [23]	83.03	—
disCLBP-TOP [23]	91.40	—
FLA [4]	86.37	—
FLA+Age [4]	92.90	—
DLM [5]	94.25	—
SID	80.45 ± 4.69	0.8465 ± 0.0500
FLA-all	82.03 ± 4.19	0.8762 ± 0.0362
AUDA	85.11 ± 3.46	0.9073 ± 0.0314
AUDA+FLA-all	86.82 ± 2.69	0.9236 ± 0.0242

**Table 10:** Scores obtained for the BBC database using different methods. For the scores quoted from other papers, an appropriate reference is provided.

Method	Accuracy [%]	AUC
SOAD [53]	75.00	—
CLBP-TOP [57]	70.00	—
CLBP-TOP+ [23]	80.00	—
disCLBP-TOP [23]	90.00	—
FLA [4]	85.00	—
FLA+Age [4]	90.00	—
DLM [5]	90.00	—
FLA-all	80.00	0.80
AUDA	90.00	0.90
AUDA+FLA-all	90.00	0.90

In addition to the cross-validation tests, each of which is performed on a single dataset, we exploited all the recordings from one database to train the FLA-all and AUDA methods to subsequently test them using the other database. The scores reported in Table 11 indicate that the performance is lower in such a scenario. The AU-wise features render very low scores for UvA-NEMO when trained with BBC (in the opposite scenario, the results are much better), and the cross-AU features allow for achieving classification accuracy of 74.35% and 70% for UvA-NEMO and BBC, respectively. Although these scores are much lower compared with when the models were trained and tested using the same databases (85.11% and 90%), they are still comparable to those rendered by the SOAD and CLBP-TOP methods (Tables 9 and 10). FLA-all is much more affected here, achieving the accuracies of only 55.25% and 65% for UvA-NEMO and BBC. The limited robustness of these methods may have two main reasons. The first one lies in a different frame rate (50 fps for UvA-NEMO compared with 25 fps for BBC) and that the videos were recorded in a different setting using various cameras (the latter may affect the tools employed to extract the AUs and localize the facial landmarks). It is worth noting here that contrary to AUDA, FLA does not take into account the frame rate during feature extraction which may make the trained model adapted to a specific frame rate. The second reason is that the criteria of creating the reference data may have been different for both databases. In the case of UvA-NEMO, the recorded subjects were stimulated in the same way—they were shown short funny videos to elicit spontaneous smiles, and they were asked to pose a smile as realistically as they could. Unfortunately, this procedure is not clear for the BBC dataset, and it is actually unknown whether the labels were defined based on how the subjects were stimulated or relying on the judgement of an expert. Overall, these scores indicate that the possibility of transferring the models across different databases (including the devices used for video acquisition) remains a challenging research problem which has not been tackled in the literature so far.

**Table 11:** Scores obtained for the UvA-NEMO and BBC databases using a model trained with a different database.

Training set →	BBC		UvA-NEMO	
Test set →	UvA-NEMO		BBC	
Method ↓	Accuracy [%]	AUC	Accuracy [%]	AUC
AUDA	66.05	0.7080	75.00	0.7400
AUDA (AU-wise)	53.06	0.5151	75.00	0.7200
AUDA (Cross-AU)	74.35	0.7780	70.00	0.6700
FLA-all	55.24	0.5566	65.00	0.6700

Overall, AUDA outperforms the SOAD and CLBP-TOP techniques, and its performance is comparable with that obtained using FLA and CLBP-TOP+ features. These results indicate that the AU dynamics standalone allow for discriminating between spontaneous and posed smiles, while preserving high interpretation capabilities. The disCLBP-TOP and DLM methods, as well as FLA enriched with the age-based stratification that exploits additional metadata (FLA+Age), perform better than AUDA. DLM and disCLBP-TOP capture information on facial appearance which is not present in the AU signals that constitute the input data for AUDA. We have published the features extracted for the UvA-NEMO and BBC datasets (available at https://doi.org/10.7910/DVN/X5QGLA), which makes it possible for the community to employ more sophisticated classification methods, as well as to combine them with appearance-based features, to further improve the performance of emerging algorithms.

## Conclusions

In this paper, we presented a new AUDA technique for capturing the dynamics of facial action units. The elaborated features were used to classify the smiles as spontaneous or posed, and we demonstrated that these features are competitive with the features extracted from the facial landmarks [4] as well as with the CLBP-TOP+ textural features extracted from spatial-temporal blocks [23]. An important benefit of our approach is that it offers interpretability in the domain of facial action units that are widely used for analyzing facial expressions. Overall, we have proved that classification of smile genuineness can be entirely based on the AUs defined in FACS. Furthermore, we proposed a new technique for identifying the smile phases from video sequences. We demonstrated that it does not require an analyzed sequence to contain a single smile cycle and it is robust against the Gaussian noise.

The experimental study has shown that although the proposed technique is highly effective, the DLM and disCLBP-TOP methods [5] render higher classification scores for the UvA-NEMO database. They are based on the features which capture the facial appearance, allowing them to extract more information on the subject (like age or gender). Our AUDA method, as well as FLA [4], are based on the data that abstract from the appearance of a subject (these are the facial action units for AUDA and facial landmark positions for FLA). On one hand, this is a certain limitation of AUDA, but on the other hand, our features can be fused with the appearance-based information at a later stage. Furthermore, our experiments concerned with feature selection using RFE have shown that there is considerable redundancy within the AUDA features. Our ongoing research is aimed at exploiting deep recurrent neural networks to analyze the dynamics of AUs, and we use the attention modules to highlight the most important features [67]. In addition to that, the networks fed with AU dynamics may be coupled with the branches with convolutional layers to extract and benefit from the appearance-based features. These research directions can be explored in the future, and for this purpose, we publish the features extracted from the UvA-NEMO and BBC databases, alongside the first-order and second-order dynamics.

The research reported in this paper is limited to the problem of recognizing the smile genuineness, but potentially the AUDA features can be exploited for solving alternative tasks related with facial expression analysis. Furthermore, it would be interesting to determine not only whether the smile is genuine or posed, but to recognize the underlying emotional state that triggered the smile. However, to train and validate such approaches, it would be necessary to create appropriate benchmark datasets in cooperation with the psychologists. Creating more datasets may also help address the problem of the model transferability across different databases which would make the methods more robust in the real-life scenarios under uncontrolled conditions.
